# Dr. Scudamore's Chemical and Medical Report on Mineral Waters

**Published:** 1821-03-01

**Authors:** 


1. *A Chemical and Medical Report of the Properties of the Mineral Waters of Buxton, Matlock, Tunbridge Wells, Harrogate, Bath, Cheltenham, Leamington, Malvern, and the Isle of Wight.* By CHARLES SCUDAMORE, M.D. Member of the Royal College of Physicians, of the Medical and Chirurgical Society of London, &c. &c. Octavo, pp. 265. London, 1820.

2. *Practical Observations on the Medicinal Powers of Mineral Waters, and of the various Modes of Bathing, &c. &c. &c. with Remarks on Exercise and Diet.* By PATRICK MACKENZIE, M.D. Licentiate of the Royal College of Physicians, and Assistant Physician to the Fever Institution of London. Octavo, pp. 216, *2d Edition, enlarged.* London, 1820.

GOOD and evil are almost as intimately blended in the moral and physical world around us, as nerve and blood-vessel in the wonderful microcosm within us. If the earth pours forth miasmata that scatter sickness and death among all within their range, she also distils from her secret recesses salubrious springs, to revive the drooping invalid, expurgate the pampered epicure, and dispel those physical sources of melancholy that sap the foundation of human enjoyment, or absolutely abridge the range of life.

The sensible qualities of mineral waters must have attracted attention at a very early epoch, and accident, of course, discovered their medicinal virtues. That they should be held in great veneration during ages of ignorance and superstition, is not to be wondered at. Pliny informs us that the ancients attached a tutelar divinity, the friend of mankind, to each medicinal spring. The moderns have converted this idea into a reality, with considerable additions and improvements. Few medicinal springs are now unattended by numerous tutelar guardians, the friends of mankind, and who, though not actual divinities, are often regarded as little less, by those who invoke their aid in the recovery of health.

It is undoubtedly of much greater consequence to ascertain, with accuracy, the *effects* of mineral waters on the human frame, in health and in the various states of disease, than to analyse their constituent principles. But to effect the former purpose, it is requisite that a man should rigidly observe and examine, uninfluenced by any of those little biases or partialities, which are so apt to insinuate themselves into the human breast, where personal interests are concerned. A physician, therefore, who visits various medicinal springs, for the purpose of reporting on their physical properties, is very likely to be impartial; but the shortness of his stay is likely to be attended by a deficiency of observation and experience. We must be contented with this mixture of good and evil here as elsewhere.

Dr. Scudamore, already so well and so favourably known to the profession, having visited several of the most remarkable watering places of this kingdom, in the autumn of 1819, was naturally led to examine the state of their mineral springs, and soon found reason to suspect the fidelity of the existing sources of authority respecting them. This again led him to an extensive inquiry into the subject, in which he had the able assistance of Mr. Garden of London. They made the preliminary experiments at the springs of Buxton, Harrogate, and Tunbridge Wells; but except at these places, the shortness of their stay did not permit them to examine the gases in the usual method. "The greater part of the waters were wholly examined as to their solid contents in London." Here, in addition to Mr. Garden's skill, our author had the advantage of that of J. G. Children, Esq. whose contributions appear frequently in the work. Dr. Scudamore's reasons for appearing before the public, on the present occasion, are, that the waters of Buxton had not been examined since 1784, and those of Harrogate not since 1794. Modern chemistry has, since these periods, afforded improved methods of analysis, and led to new reasonings on the medicinal properties of mineral waters.

After some preliminary observations on chemical tests, our author commences with BUXTON.

§ 1. This far-famed resort of invalids is a considerable village in Derbyshire, distant 159 miles from London, the surrounding country being a wild mountainous district, thinly inhabited, and presenting a rude character of scenery. The village itself is situated in a valley surrounded by hills. The Buxton mineral water rises freely by numerous fissures through the limestone, and is conducted from the springhead, by an artificial sandstone channel, into a large marble basin, open to the air in front, and secured from intrusion by an iron gate. On emerging from the earth, its temperature is 82°—in the basin 77°. The water is transparent, odourless, tasteless—affecting neither litmus nor turmeric paper. Specific gravity 1.0006, at the temperature of 60; but at the spring head, 999°. The action of re-agents led Dr. S. to the conclusion, "that this water contains muriatic acid and sulphuric salts with bases of lime and magnesia, in small proportions." 12. Analysis of the gazeous and solid constituents developed the following substances in one gallon of the water; viz. carbonic acid 1.50 cubic inch—azote 4.64 ditto; muriate of magnesia .58; muriate of soda 2.40 grs. sulphate of lime .60; carbonate of lime 10.40; extractive matter and vegetable fibres .50; loss .52; total 15 grains.

*Medicinal Qualities:* As an internal remedy this water is not so celebrated as the baths externally; yet their reputation is considerable. From the very small proportion of ingredients, some physicians have denied these waters any other virtues than those of common warm water; but, as Dr. S. justly observes, the state of very minute division in which the substances are, may enable them to act with considerable energy on the sensible surface of the stomach, when empty, and thus be rapidly absorbed into the torrent of the circulation. Nevertheless, Dr. S. is induced to believe, that "the medicinal action of Buxton water must be referred to its purity, its temperature, and its gazeous impregnation with azote." 19.* Simple as it appears in composition, it occasionally proves inconveniently stimulating to some invalids of full habits and sanguineous temperament, who complain of flushing, headache, and slight giddiness that deter them from proceeding. In Dr. S's experience, this water has excited gout. In general, one or two doses of aperient medicine should precede the use of the water; and arthritics should not begin a course of it until they are freed "from every discoverable sign of an active state of the gouty diathesis."†

The first dose of the water (about half a pint) should be taken an hour before breakfast, and where the bath is used, after, not before, that operation. The dose should be taken

* "Buxton water is remarkably pure, and differs from common spring water only in its temperature; and the small quantity of azotic gas which it holds in solution." *Mackenzie, 2d Ed. p.* 37.

† Dr. Mackenzie remarks, that "it appears to produce various effects on the bowels—not unfrequently a diarrhœa, or looseness succeeds, for a few days, its use." *2d Ed. p.* 39.

at twice, with the interval of a quarter of an hour, using moderate exercise the while. At 12 or 1 o'clock, the dose may be repeated in the same way. In a week or ten days, the total quantity, *per diem*, may be increased to a pint and a half. There is rarely any advantage in proceeding beyond that quantity. If the water prove too stimulating, it should be discontinued for a time, and proper means taken to reduce the inflammatory excitability of the habit, after which it may be resumed.

"When the water agrees perfectly well, it sits pleasantly on the stomach, is refreshing, by degrees produces a sensible improvement of the appetite, assists the digestion; and, thus amending the functions of the stomach, conduces to the general strength of the body, and consequent cheerfulness and comfort of mind. In concluding with a general medical character of the water, I may affirm that it proves very generally beneficial to the dyspeptic patient; and that it is a valuable auxiliary to the use of the baths. In the condition of stomach which gout produces, and also in the state of constitution which is associated with chronic rheumatism, the internal use of the water has in many instances within my knowledge afforded decided benefit; and, therefore, although it be less sensibly active in its properties than some of the other waters of which I treat in this little volume, it deserves, I am persuaded, to be regarded as considerably medicinal and useful." 23.

At Buxton are constructed cold, warm, and vapour baths, with every degree of convenience, except in ventilation and dressing apartments, of which our author complains. The temperature of the public and private baths is 82°. A considerable quantity of free azotic gas rises in bubbles to the surface of the baths.

Before the invalid enters on the use of the bath, he should adopt some preliminary preparation. If, for instance, there be marks of congestion about the head, cupping or leeching may be proper. If the tide of the general circulation be increased, venesection will be more appropriate. Some suitable aperient medicine should also be premised, and the alvine excretions constantly attended to afterwards. Mercurial medicine should, of course, be avoided during the use of the bath. "The temperature of 82° is not sufficiently high to favour that action of the skin which conduces to the safe and favourable action of mercurial alteratives." 29.

"The class of patients resorting to the Buxton bath comprise, for the most part, those who have suffered either from gout or rheumatism. But it is by no means equally proper for the gouty and the rheumatic invalid under circumstances apparently similar. I should forbid the use of the bath to a patient actually suffering the pains of chronic gout; and I should consider him to be requiring suitable medicines to remove such a diathesis, as an essential preliminary. The bathing will be a valuable remedy to relieve that debility of limbs, and of the whole constitution, which is a common sequel to chronic gout, and which seems to partake very much of the character of rheumatism. When gout, from the frequency and severity of its attacks, has not only debilitated the limbs in a serious degree, but has also weakened the constitution, so that the circulation is very languid, and the nervous system much depressed, it appears to me that a course of warm sea-bathing, sea air, and friction, should precede the visit to Buxton; or, if circumstances do not allow this arrangement, the warm bath at Buxton may be the previous remedy, the temperature being gradually reduced to prepare the patient for that of 82°." P. 30.

When any degree of acuteness is perceptible in rheumatism, our author thinks the bath of Buxton improper or actually injurious. "But flying pains, with a natural state of the pulse, do not constitute an objection." It is where the various textures concerned in muscular motion are weakened, attended with lameness, stiffness, and irregular pains, particularly in damp weather, and before atmospherical changes, that the Buxton bath produces the happiest effect.

Excepting where there is much constitutional debility, the patient should bathe before breakfast, and that by *plunging* in. The stay, at first, should not exceed one or two minutes; and if a pleasant re-action or universal warmth succeed, the bath agrees; and *vice versa*. Dr. Scudamore is convinced that the good effects of this bath would be greatly enhanced by friction or shampooing put in force immediately after leaving it.

Our author praises the air of this place, as remarkable for its bracing qualities. Although its hilly site renders the climate variable, the rains are quickly absorbed, and the atmosphere is comparatively dry. Neither is the vicinity devoid of interesting scenery, and the invalid, at little expence or trouble, may see the "seven wonders"—not of the world, but of the Peak.

Besides the tepid springs of Buxton, there is also a chalybeate, of weak impregnation, but which, when necessary, may be strengthened by the tinctura ferri muriatis.

§ II. Matlock, 22 miles south-east of Buxton, situated in a valley close to the river Derwent, is overhung by mountain scenery of the highest order of picturesque beauty. Its fountain differs little in sensible properties from common spring water, except in temperature, which is 68°; hence, in taste, it approaches to tepid. It is beautifully clear, but not very sparkling: its specific gravity at 60°, being 1.0003. From the effect of re-agents, our author concludes, that the water contains free carbonic acid, and some muriates and sulphates in minute proportion, whose bases are probably magnesia, lime, and soda.

"I do not feel authorised to extol the water as a relief for any particular class of disorders. Its purity, its agreeable temperature, and its freshness, ensure to the invalid that, while it has the chance of being in some measure useful, it will not disagree. The immediate impression on the stomach is more grateful than that occasioned by ordinary spring water; is more tonic; and when attention to regimen is joined with a course of it, much decided advantage may be expected." 44.

There is a natural bath at the temperature of 68°, which is just intermediate between Buxton and the sea. It produces a slight shock, less than the cold bath, and which is soon followed by a re-action, and an agreeable glow. It is inefficient in rheumatism, and for the most part "inapplicable for a gouty person." 46.

§ III. Tunbrige Wells. This sectiou is almost a literal reprint of Dr. Scudamore's former publication on these waters in 1816. In order not to break the chain of our analysis, we shall consider this section in the same manner as the others. This fashionable watering place is situated in the weald of Kent. The spring rises into a large marble basin, overflows into a channel connected with the chalybeate cold bath, and, in its progress, deposits a reddish brown precipitate. The fresh water is perfectly transparent, and without air bubbles, exhaling a distinctly chalybeate odour, with a strongly marked taste, neither acidulous nor saline, and by no means unpalatable. Our author's experiments lead him to conclude, that the spring rises from a great depth—that it contains iron, in probably no very slight proportion—that this iron is combined with carbonic acid—that free carbonic acid is contained in the water, together with a carbonated earth, lime, combined sulphuric acid, and muriatic salt. The water appears to contain about 7½ grains per gallon of ferruginous and saline ingredients. In respect to its gazeous contents, it was found to present per gallon, 8.05 cubic inches of carbonic acid—.50 of oxygen—4.75 of azote.

The chalybeate spring, behind the Sussex hotel, contains little more than a grain of iron in the gallon of water.

*Medicinal Properties.* Dr. Saunders observes, that "the most noted of the simple chalybeates in this country is that of Tunbridge Wells." Dr. Scudamore adds, that

"The mildness and salubrity of the air, joined with the remarkable beauty of scenery in the surrounding country, render Tunbridge Wells a situation of resort for the invalid at once valuable and delightful." 71.

The very minute proportion of iron in these waters has induced some people to be sceptical as to their powers. But medical and chemical writers agree that, the most active form in which iron can be administered as a medicine, is in the state of solution by carbonic acid. Dr. S. has shewn, that the iron continues in perfect solution in this water, at a temperature a little beyond 140°, a heat full 40 degrees higher than that of the human stomach. Hence, he thinks that, in this state of perfect chemical activity, it exerts its agency in a very direct manner over the whole surface of the stomach to which it is applied. He thinks it probable, also, that the mineral may partly find its way into the torrent of the circulation, in its entire state of solution. The action of the carbonic acid and the azote is also to be considered.

On all occasions some aperient medicine should be premised, after which, the patient is to take the first dose at 7 or 8 o'clock in the morning; the second at noon; and the third about 3 in the afternoon. The quantity must vary with the case; but it is best to begin with small doses, taking in all, from half a pint, to two pints daily.

The principal diseases to which the Tunbridge waters are applicable, are dyspepsia, from debility of the stomach, with languor and nervousness—uterine debility—chlorosis—cutaneous complaints, especially of the scaly species, connected with weakness of stomach—gravel, founded on unhealthy condition of the digestive organs.

"Its general operation is to increase, in a gradual manner, the tone of the secretory system, and, by the permanency of its tonic power, to augment the strength, nervous energy, and vigour of all the functions of the body. It is, therefore, in those chronic diseases that arise from slow beginnings, and are attended with great laxity and debility of the solids, that this water is particularly indicated." *Mackenzie, 2d. Ed. p.* 69.

Dr. Scudamore considers the most favourable period for employing the waters of Tunbridge Wells, to be from May to November.

§ IV. Harrogate, is situated agreeably in the centre of Yorkshire, 211 miles from London, the whole neigbouring district abounding with mineral springs, principally sulphuretted and chalybeate.

From the Old Sulphur Well, the only one now resorted to for drinking, the water rises at first transparent, with a very strong sulphureous and fetid smell, which has been compared to the washings of a rusty gun-barrel. The taste is saline and disagreeable. If this water be bottled at the spring, and immediately corked and sealed, it retains its gas and all its virtues for a long time—even for months. The temperature of the water is 54°. The action of tests led Dr. Scudamore to presume, that it contains muriatic and sulphuric acids, united to lime and magnesia, with a strong impregnation of sulphuretted hydrogen gas. The portion of gas insoluble in water, consists of carburetted hydrogen and azote, in nearly equal volumes. Dr. S. and Mr. Garden give the following composition in one gallon of the water:—

*Gazeous Contents.* Sulphuretted hydrogen, 13.716 cubic inches—carbonic acid, 9.529—azote and carburetted hydrogen, 5.800—total, 29.045 inches.

*Solid Contents.* Muriate of soda, 760 grains—muriate of lime, 32 grs.—muriate of magnesia, 28 grs.—sulphate of lime, 8 grs.—carbonate of lime, 12 grs.—carbonate of magnesia, 3 grs.—loss 5 grs. Total, 848 grains.

*Medical History, &c.* Previous to using the Harrogate waters, some preparatory treatment is generally necessary. The plethoric should lose a few ounces of blood, either from the arm or by cupping; while a mercurial cathartic should be administered, to clear the primæ viæ. When there is indication of a sluggish circulation in the abdominal organs, or congestion in the portal circle, a course of eccoprotics, as the blue pill, and aloetics, every second night, should be enjoined. In general this water is not sufficiently aperient of itself, and therefore, requires an auxiliary. The patient should rise early, and drink the waters fresh from the spring. The medium dose is three quarters of a pint, at two draughts, interposing some exercise between them. The whole to be taken before breakfast. "Sugar comfits and aromatic seeds, are frequently eaten to correct the nauseous taste of the water; but Dr. Garnett recommends a small quantity of sea-biscuit or coarse bread." *Mackenzie, p.* 129. A full course of the waters requires a period of from four to six weeks. As the water may be taken to any distance, the patient may continue or resume the use of it at home, afterwards.

Dr. Scudamore thinks, that the Harrogate water is, every year, gaining reputation in hepatic diseases, and intestinal torpor, in conjunction with the blue pill and ex. col. comp. Dr. Garnett recommends injections of the water *per anum.*

"Of cutaneous diseases, it is in the order squamæ of Willan, and the species lepra and psoriasis, that Harrogate water promises the most benefit. Dr. Willan gives his valuable testimony to its efficacy, when he remarks, 'I have seen some very obstinate cases of lepra, alphos, and psoriasis, completely cured by the proper use of the waters of Harrogate.'" *Scudamore*, 105.

Dr. S. does not think the water is likely to prove very successful in the different kinds of *acne*, while in the species rosacea, or gutta rosea of authors, it is apt to prove decidedly hurtful. Dr. Armstrong, in his valuable work on Scarlet Fever, is well known to speak very highly of Harrogate water in various diseases of the skin, joints, and viscera. Dr. Scudamore thinks it would, in all probability, be very advantageous in gravel, and habitual deposition of lateritious sediment in the urine.

In respect to the Harrogate *bath*, Dr. Scudamore thinks, that the same general principles which regulate the use of the ordinary warm bath are applicable to it, with some additional cautions. For instance, the same preparations should be enjoined that precede the internal use of the waters; and as the bath is a considerable stimulant on the skin, it should not be taken while any feverishness obtains in the system. Dr. Garnett thought, that the water was absorbed by the skin. Without fully acquiescing in this principle, Dr. Scudamore concludes that, "its strong impregnation with saline and gazeous matters, causes it to act very decidedly on the sentient surface of the body, and indirectly, by sympathy, on the internal organs." 109.

People with cutaneous complaints should use the bath shortly before going to bed, and drink some warm diluting fluid afterwards. In other disorders, the bath may be taken an hour or two before dinner. The stay in the bath will vary from ten minutes to twenty-five. The bath is to be considered as an auxiliary remedy in those diseases where the water is taken internally. It is calculated to afford considerable relief in the sequelæ of gout and rheumatism; but is inadmissible when decided gouty action, or rheumatic inflammation is present.

Oddy's Saline Chalybeate Spring is of considerable importance. It contains magnesia, lime, and iron, combined with muriatic, sulphuric, and carbonic acids. The solid contents in a wine gallon are, 300 grains of muriate of soda —22 of muriate of lime—9 of muriate of magnesia—6.7 of carbonate of lime—1.86 of sulphate of lime—2.40 of oxide of iron.

The properties of this spring are alterative and tonic in a high degree. It appears to Dr. Scudamore to be a water possessing "an excellent combination of saline ingredients, and of oxide of iron, held in solution by carbonic acid." 120. Our author offers the same general rules, for the exhibition of this water, as for that of Tunbridge Wells.

Close to the above spring is another, and simply chalybeate water. Of this we gave an account in the 8th No. for April, 1820, p. 650. Dr. Scudamore doubts the correctness of Dr. Adam Hunter's statement, that this spring contains ten grains of carbonate of iron in the gallon. He makes it only one grain and a half. But Doctors will differ on more subjects than those of mineral waters.

Harrogate and its vicinity can boast of much interesting scenery, and the air is bracing to the invalid whose case may require "sulphuretted and chalybeate springs of superior pretensions."

§ V. Bath. The mineral springs of this celebrated place are the only ones we possess, which may be called hot to the touch. They appear to be all derived from the same source, varying in their temperatures, in consequence of their course to the surface being more or less circuitous. Their chemical impregnations vary only in degree. The King's Bath water yielded the following saline contents, according to the analysis of Dr. Scudamore and Mr. Garden. In a pint of the liquid were found 1.2 grs. of muriate of lime—1.6 ditto muriate of magnesia—9.5 ditto, sulphate of lime—.9 ditto sulphate of soda—.01985 ditto, oxide of iron—1.2 cubic inches of carbonic acid.

Our author thinks, that the magnesia in these waters (which he has discovered) is one of their most important ingredients. The King's Bath water is more strongly impregnated with magnesia than the Cross Bath, but not quite so much so as the Hot Bath. The following remarks are in reference to the King's Bath Pump. In judging of the medicinal properties of this water from Mr. Phillips's analysis, by the direct mode, one would be led to attribute very little influence to it, excepting as a slight chalybeate:—

"But upon Dr. Murray's views, the water will acquire higher pretensions. In his calculation, he gave to a pint of the water 3.1 gr. of muriate of lime,* and raised the proportion of sulphate of soda from 1.5 gr. to 5.5 gr." 141.

"* Dr. Murray did not know of the existence of magnesia in the water. If the whole of the muriatic acid were supposed to be combined with lime, the muriate of lime would be almost accurately 3.1 grs.; according to my present analysis."

Dr. Murray exaggerates the efficacy of the muriate of lime, and was ignorant of the existence of magnesia in the waters The quantity of iron is so small, that unless we view it as a protoxide held in solution by carbonic acid, we cannot easily account for its effects on the human body. Here, our author, as indeed, in most other parts of the volume, offers an epitome of what others have written on the subject of Bath waters, with such comments of his own as his limited experience and general reasonings enable him to make. Dr. Falconer recommends these waters in cases of deficient nervous energy, usually stiled cachectic, as chlorosis, visceral obstructions, "that hardness about the region of the liver and spleen which often succeeds to intermittent fevers," &c.

"Sir G. S. Gibbes advises the use of the water in that condition of the liver in which its functions are remarkably inert from obstruction, unattended with any inflammatory action, and when the stomach is affected with dyspeptic symptoms, dependant upon general want of tone in the digestive organs. A degree of jaundice attends this state of disorder, and the stimulus of the water has the praise, from these authors, of exciting healthy action in these important viscera, in a remarkable manner." 147.

Dr. Scudamore very properly questions the propriety of employing the Bath water in visceral obstructions, since it is difficult to imagine obstruction in an organ, independent of some degree of inflammation or congestion, when the stimumulant effects of these waters would be injurious.

"I would say for the most part, the Bath water should not be employed in complaints of the abdominal viscera, while any absolute obstruction is actually existing. As a tonic remedy, after the sufficient employment of regular medicines, it is entitled to our best confidence. It is always to be considered in diseases of obstruction, that if we stimulate the organs of circulation prematurely, it is more probable that we shall excite diseased, than healthy action. It must be our object to restore proper function before we can, with any fair prospect of advantage, excite the unhealthy organ or organs, to a greater degree of action;—if action alone can be considered." 148.

In *paralytic* affections, it is obvious, that great care is necessary in prescribing these waters, now that the pathology of palsy is better understood than formerly. In dyspepsia, resulting from studious and sedentary habits, these waters, Dr. S. thinks, may be very generally applicable. In respect to gout, a subject on which our athor will be listened to with attention, these are his sentiments.

"I speak from sufficient experience in saying that the Bath water, either employed internally or externally, is inadmissible, when an active state of gouty diathesis is present; when the tendency to relapse is strongly established in the constitution, whether from the use of Eau Medicinale, Wilson's Tincture, Reynolds's Specific, or similar baneful medicines; or from continued irregularities in living. Also, when plethora; a state of circulation easily excited to inflammatory action; or marked obstruction in the vessels of the liver, are found to exist. As a general opinion I would venture to observe that a gouty patient should be restricted to any free use of the water, and perhaps to its employment altogether, unless debility of the stomach, or nervous system, unattended by gout, prevail; or unless that kind of chronic gout is happening in which it is to be desired that a fit, as it is called, should be excited for the relief of the constitution, which, under such circumstances, is oppressed with all the distressing symptoms of hypochondriasis. I must add, however, that cases of this description must be attentively studied, as to the question of visceral obstruction." 151.

Dr. S. suggests, when the Bath water is found too stimulant, to convert it into an alterant by exposing it for a few hours to the open air, and afterwards warming it to any required temperature, in which case it will have lost its oxide of iron, and some of the carbonate of lime, retaining the muriates, in full proportion, "on which so much of its virtues may be stated to depend." The *medicinal* virtues of these waters, internally and externally applied, are principally taken from Dr. Falconer and other preceding writers, consequently we cannot dwell upon them here. Dr. Scudamore thinks that it is in the chronic forms of gout, where there is great deficiency of nervous energy in the muscles, joined with languid circulation in the extremities, and stiffness with aching pains in the joints upon every motion, that we are to expect most advantage from the Bath waters, internally and externally. The dry pumping, he thinks, should be cautiously employed, and never when there is any tendency to inflammatory action in the parts. In chronic rheumatism the baths are highly useful, though not a remedy of universal application in this complaint. It will be found most efficacious in those forms where the inflammatory diathesis is absent, and in which there is but little tendency to febrile irritation. But in gout and rheumatism Dr. Scudamore does not "lose sight of the superior pretensions of the Buxton bath for most conditions of these complaints."

"Bath, I apprehend, deserves the preference only in that state of the limbs, in which the circulation is very languid, as shewn by coldness of the extremities; and remarkable stiffness constantly prevails." 169.

§ VI. Cheltenham. It is to be regretted, that some of the medical gentlemen now, and long resident at Cheltenham, should not have favoured their brethren with some account of these celebrated waters, and the diseases to which they are peculiarly applicable. It cannot be expected that a rapid visit, or a short sojourn at such a place, can be productive of much information. Into the minute details of analysis, partly original, partly compiled, which Dr. Scudamore has presented, we cannot enter here. "It is evident," says Dr. S. "from the comparative view of these analyses, that the waters in the course of the last few years, have undergone considerable changes." Thus the spring No. 1. now scarcely contains any chalybeate impregnation—while the spring No. 2, which, in 1817, was said to be more strongly impregnated than the powerful water of Harrogate, produces only a white precipitate with the acetate of lead.

"I am compelled therefore to give the following character of these waters. No. 1, a saline aperient alterative water, containing a very slight impregnation of iron, so as not to be objectionable on this account, except with a patient to whom this ingredient is forbidden even in small quantity.

"No. 2, of much the same power as No. 1, except that it has less of the muriates of soda and magnesia. It does not appear to be more strongly chalybeate than No. 1.

"No. 3, equally aperient with No. 2; has less of muriate of lime, and more of muriate of magnesia.

"No. 4 is a saline water, which appears to be wholly free from iron, but contains a good proportion of all the saline ingredients.

"No. 5 is a water not shewing any sulphuretted impregnation. It is only slightly chalybeate. It possesses a fuller impregnation both of aperient salt, and of the most important muriates, than the other waters.

"No. 6 contains only a minute proportion of iron. It is less aperient than No. 4, but contains rather more muriate of magnesia." 188.

In taking a general review of the composition of all these waters, Dr. S. finds that there are three kinds, all saline, aperient, and alterative; some containing a very feeble sulphuretted impregnation; others a small portion of oxide of iron held in solution by carbonic acid. The aperient agent is sulphate of soda—the alterative, the muriates of lime and magnesia. It is the practice to increase the purgative power of Thompson's No. 4, by the addition of a solution of the salts obtained by evaporation of the water.

We shall not dip into the dispute between Dr. Adam Neale and the proprietors of certain springs at Cheltenham, but proceed to the *medical history* of the waters, though we can expect but very limited observations from the work under review. Dr. Scudamore thinks, and no doubt with justice, that the important and salutary effects of the Cheltenham waters consist in this:—

"That an invalid can pursue a continued daily course, such as produces a regular and considerable action on the bowels, without suffering that debility of the constitution and impaired appetite, which are apt to occur from a similar course of saline aperients at home." 200.

A course of Cheltenham waters should not be entered upon without medical advice.

"As a general rule, a mercurial purgative should precede the use of the water. It is an important fact, that if much confinement of the bowels have prevailed, and more especially if there be decided biliary obstruction, the water, instead of becoming the ready remedy which is expected, may prove a source of evil in the way I shall state. It may act upon the exhalent vessels of the alimentary canal, so as to produce only fluid discharge, and actually leave behind the more solid and obstructing matter. The same observation applies in a great degree to the use of the water in progress. It is, I know, the medical practice at Cheltenham, and very judiciously, to conjoin the use of a purgative alterative pill with the water. This will of course be more or less active in its composition, according to the constitution of the patient and the nature of the case." 201.

Dr. Scudamore shortly enumerates the principal diseases to which these waters are applicable. "The gouty patient may drink the pure saline waters, with almost certain prospect of advantage." A paroxysm of the disease very commonly occurs in a short time after commencing the waters—resulting, Dr. S. thinks, "from the stimulating qualities of the muriates." In such circumstances the Doctor advises a discontinuance of the waters, during the active symptoms of gout. The fit, he thinks, does not very soon return, after an accession of this kind, provided the case has been properly managed.

The disordered condition of the digestive organs produced by residence in tropical and other fervid climes, "rank foremost in the class of Cheltenham cases."

"It is an encouraging consideration, for those who labour under disordered functions of the liver, together with debility of the constitution, that the action of the Cheltenham water on the bowels, from day to day, is not attended with the weakening effect which is liable to happen from ordinary medicine; and as the individual who has resided in a tropical climate, most usually has undermined the real powers of his constitution, this is a point of great moment.

"Every practitioner must have met with cases of diseased liver, accompanied with such an impaired state of constitution, that any active employment of mercury would be an unwise if not a hazardous treatment. It is not safe to raise up mercurial fever in the system in these instances; and I do not believe that a better expedient can be adopted, than a course of Cheltenham saline water in conjunction with a mild mercurial alterative." 205.

As an auxiliary, the warm bath, upon a regulated plan, will materially assist the alterative action of the water. With which view the patient should bathe an hour or two before dinner, and not take any unnecessary exercise afterwards. We do not think that in any medical work, not destined exclusively for the non-medical reader, such details as the following can have a very pleasing effect on the professional mind.

"As a general rule, I should class in the prohibited list, salt meat, pork, fat and skin of meat, rich made dishes, the fat part of salmon, stewed eels, lobsters, pickles, and salads; spinage, as being a vegetable which readily ferments; any vegetable which is not quite in season, sweet, tender, and well boiled; pie crust, and all rich confectionary: strong cheese, and such as is either very new or very old." 208.

§ VII. LEAMINGTON, situated two miles East of Warwick, and 90 miles from London, formerly an obscure hamlet, now assumes, every day, more and more the pride and magnificence of a modern town. The surrounding country is agreeable, and affords the invalid great variety and facility of exercise.

"Interesting objects of curiosity in the neighbourhood are not wanting. That ancient and most noble structure, Warwick Castle; the romantic attraction of Guy's Cliff; the venerable ruins of Kenilworth Castle; Stratford-upon-Avon, at an accessible distance, the well-known birth-place of our divine Shakespeare; may be mentioned as assurances to the visiter, that in pursuing his daily exercise he will find an ample share of gratification." 215.

Our author gives analyses of nine different, or at least distinct springs at this place, for which details we must refer the curious to the work itself. We can only glance at the prominent chemical and medicinal properties of a few of them.

I. *Royal Pump Room*—Saline water. This has only a small trace of iron, and therefore may be considered as an almost purely saline water, strongly alterative and considerably aperient. It contains much muriate of lime. In general, the action of the water on the bowels ought to be assisted by pills taken on the preceding night.

II. *Royal Pump Room*—Sulphur water. This is an excellent alterative water, mildly aperient, containing very good proportions of muriates of lime and magnesia, and an active impregnation with sulphuretted hydrogen. The oxide of iron is very trifling, yet sufficient to increase the stimulant property of the water. It does not keep, like the Harrogate water, but loses all its sulphuretted hydrogen.

III. *Lord Aylesford's Springs.* This water is considerably aperient and alterative, abounding in muriate of lime, and containing very little iron.

IV. *Marble Bath Pump*—right urn. Nearly resembles Harrogate waters in its impregnation with sulphuretted hydrogen, and, in its saline contents, is a stronger water than that at the royal pump room. The *left urn* is a very strong chalybeate alterative.

"The waters of Leamington, as compared with those of Cheltenham, are, according to my view of their comparative composition, considerably different in their medicinal character. The saline class are much more highly impregnated with muriate of lime; the sulphuretted in the one instance powerful, and the other almost negative; the chalybeate of very superior activity. But it does not follow that the invalid should, from this statement, give a necessary preference to the springs of Leamington. On the contrary, in all these cases in which the most saline; or, in familiar language, the most cooling aperient waters are required, Cheltenham will deserve the preference. In general terms, I am disposed to consider that the use of the waters of Cheltenham should sometimes be introductory to those of Leamington; as being less stimulating." 234.

§ VIII. *Malvern Waters.* The village of Malvern is situated eight miles from Worcester, and its waters are celebrated for their purity. Dr. Scudamore having very little experience of these waters himself, gives the description of them from Dr. Saunders' treatise. He is not inclined to attribute to waters of so remarkably slight impregnation, those varied virtues pourtrayed by Dr. Saunders.

"This is certain.—The salubrious air of Malvern, and the peaceful feelings which the quiet and charming retirement of the spot inspires, contribute in the greatest degree to strengthen the body, to calm the mind, and thus to promote the general health. It is from such a conviction, that I have advised the Cheltenham invalid to repair to this favoured situation, at a certain period after the use of the aperient alterative waters."* 245.

---

* Dr. Scudamore, in alluding to Dr. Philip's analysis of these waters, observes that he could not detect iron in them, and that he found the water of Holy Well and that of St. Ann's "precisely similar." Dr. Philip has recently replied to this observation in the London Medical Repository for December, shewing that his analysis was made with the greatest care "by repeated distillations of a gallon of each of the waters

It only remains for us to notice the "aluminous chalybeate spring" in the Isle of Wight, the account of which is entirely taken from Drs. Marcet and Lempriere, whose writings on the subject have been long before the public. Upon Dr. Murray's principles of analysis this spring would appear to contain, in each pint, 41 grains of sulphate of iron—31 of sulphate of alumina—7 of sulphate of lime—2 of muriate of lime—1 of muriate of magnesia—22 of sulphate of soda. From this view, the water must possess great medicinal powers—the sulphate of iron being (next to the muriate) the strongest of the salts of that metal. The muriate of lime is in efficacious quantity; while the sulphate of soda will tend to obviate the restringent action of the water on the bowels. We shall terminate this article with an extract from Dr. Lempriere's work.

"In administering the water, it was a rule, previously to devote one day to clearing the bowels by a suitable aperient; and the sulphate of magnesia, or Epsom salts, was the medicine generally preferred. Under this preparation, the water seldom produced any disagreeable effect on the stomach or bowels, or rendered it necessary, during the course, to take laxative medicines; an advantage which does not attach to the other chalybeate waters, unless they hold in solution a considerable portion of some aperient salt.

"From the active substances contained in the aluminous chalybeate water, Dr. Saunders, as well as Dr. Marcet, have very judiciously recommended, that, in the first instance, it be diluted. To patients with delicate stomachs, or in irritable habits, this precaution, as well as that of taking off the chill by immersing the glass in warm water, seems adviseable; but in the Walcheren cases, the only qualification the water received, was the addition of a drachm, or teaspoonful of the compound tincture of cardamoms, to each dose, which at first was only two ounces, or a small wine glass full; and this was repeated three times a day, giving the water at those periods which would the least interfere with the hours of meal. When first prescribed, it was thought adviseable that it should not be taken in the morning fasting; but in this, as well as in many other particulars, the practitioner must act, as circumstances shall suggest, bearing in recollection, that tonic medicines, in general, produce the greatest effect upon an empty stomach.

in closed glass vessels, and without allowing the water to boil;" having found, from several trials, that such a proportion of the solid contents as considerably influences the result, where the whole quantity is so small, as in the Malvern waters, passes over when the distillation is performed by boiling. He thinks it needless to state that it was impossible for him, on comparing two powders, to mistake a whole for a half—"for the solid residuum of St. Ann's water was never more than about one half of that of the Holy Well water." *Vide Med. Repos. ut supra.*

"In about three days, the dose of the water was increased to three ounces, or a larger wine glass full, with the same proportion of tincture of cardamoms, three times a day; and at intervals, it was thus gradually augmented, until a pint, in four doses, could be taken in the twenty-four hours, though, in most instances, twelve ounces, or three quarters of a pint, were found sufficient.

"The water, no doubt, might occasionally be given without the tincture of cardamoms or any other addition; but independently of the risk which would thereby be incurred, of nauseating the stomach, it seems to have derived considerable efficacy from being combined with an aromatic; in the choice of which, the practitioner must be regulated by the habits and constitution of the patient, as well as by the particular case thus brought under his consideration.

"In a course of this water, costiveness, which, with me, the remedy seldom induced, is *most particularly* to be guarded against, by the occasional use of a suitable aperient, of which the sulphate of magnesia, or the aloetic pill with myrrh, was generally preferred; and a laxity of the bowels, if it extends beyond a *temporary* effect, may easily be restrained by adding to each dose a few drops of the tincture of opium, or, if further necessary, by qualifying it with some aromatic astringent." 261-3.

We believe that we have now presented as faithful and concentrated an analysis of Dr. Scudamore's volume, as could well be comprised within the limits of this article. And having done so, we do not conceive that we are called upon for any opinion or comment as to its general merits, since our readers are put in possession of ample documents on which to ground their own judgment in the case.

Dr. Mackenzie's little volume, being a professed compilation, has not been brought forward analytically; and therefore, in justice to its merits, we consider ourselves bound to say that it is a very useful little work, embracing much interesting information in a narrow compass.

